# An Introductory Synthetic Data Tool

**DOI:** 10.23889/ijpds.v8i2.2255

**Published:** 2023-09-14

**Authors:** Iori Thomas, Bobby Stuijfzand

**Affiliations:** 1 Behavioural Insights Team, London, United Kingdom

## Objectives

Synthetic data reproduces features of a dataset without disclosing sensitive information, allowing researchers to explore data structures and test code without requiring access to real, potentially sensitive, data. We produced a low-fidelity synthetic data generation tool, accompanied by extensive documentation, allowing novice and expert users to produce such data.

## Method

Our tool, consisting of a Python notebook and a user guide, takes a dataset as input, and produces ‘low-fidelity’ synthetic copy of this dataset, recreating the data fields (or columns) of a dataset, as well as the data types and statistical relationships within these fields, but not between them. It has been tested using real-world administrative data sets and with several users, looking at the quality of the data generated, inspecting whether the data is indeed low-fidelity (i.e. statistical relationships between fields are not recreated) and the usability of the tool.

## Results

Our tool successfully created synthetic datasets from administrative datasets. Users were positive about its usability and the generated data. Tests indicated that computational memory is a main constraint on the size of datatable that can be read in by the tool. We have since implemented improvements to the memory efficiency of the tool to partially address this and have also added procedures that allow for using subsets instead of complete datasets, allowing for the use of datasets which would have otherwise been too large to be used. Testing further indicated that, while the tool by design does not preserve any relationships between fields, they can be reproduced by coincidence, and a limited disclosure process may be required when correlations from the original data are reproduced.

## Conclusion

The tool is easy to use and therefore a useful introduction to synthetic data, providing users with a foundation before using more sophisticated synthetic data tools like Synthpop. Future work could include the development of a Python library and extension of the tool to handle linked datatables.

